# Artificial Empathy in Therapy and Healthcare: Advancements in Interpersonal Interaction Technologies

**DOI:** 10.34133/cbsystems.0473

**Published:** 2025-12-16

**Authors:** Tianyu Jia, Feiyu Pan, Xingchen Yang, Linhong Ji, Dario Farina, Chong Li

**Affiliations:** ^1^ Department of Bioengineering, Imperial College London, London W12 0BZ, UK.; ^2^ Department of Mechanical Engineering, Tsinghua University, Beijing 100084, China.; ^3^ School of Automation, Southeast University, Nanjing 210096, China.; ^4^ School of Clinical Medicine (BTCH), Tsinghua Medicine, Tsinghua University, Beijing 100084, China.

## Abstract

The healthcare sector is challenged by critical workforce shortages, and this is causing an urgent need for innovative technologies to support or augment human roles. Although much of the research effort has focused on support and training of functional tasks, the emotional impacts that humans bring to the loop have often been overlooked. This gap is particularly pressing in healthcare and therapy, where empathy and emotional support are central to patient well-being. Unlike machines, humans possess a unique capacity for empathy, connecting emotionally with others and providing the essential support that fosters healing. Bridging this gap requires integrating affective elements, such as empathy, into therapeutic systems, which is the key to improving their effectiveness. This review explores groundbreaking techniques that integrate interpersonal interactions within therapy and healthcare, focusing on multiplayer games that strengthen real-time social connections, alongside social robots and virtual agents designed to simulate human-like affective interactions. Using artificial intelligence, these technologies aim to replicate complex human dynamics and foster artificial empathy, thus revolutionizing how we deliver care and support.

## Introduction

The global healthcare sector faces a progressively worsening workforce shortage. According to the World Health Organization (WHO), there may be a shortfall of up to 10 million healthcare workers within this decade, including doctors, therapists, and nurses [1]. In the future, the widespread application of technological advancements is an inevitable trend to counteract the growing need for healthcare and therapy for the aging human population. Technological advances are increasingly addressing these shortages by automating routine tasks and improving the efficiency of healthcare delivery. For instance, robotic systems now assist with diagnostics, treatments, and daily care tasks, reducing some burdens on healthcare professionals. However, while robots excel in tasks such as replicating rehabilitation movements with high repeatability and standardization, they have not yet matched the effectiveness of human therapists [2]. One important missing factor in robot-based training is the positive affective effects on motor relearning that usually come from the training through interpersonal interaction led by rehabilitation physicians. This factor remains largely underestimated, and integrating it into technological systems remains a major challenge to further improve robot-based training.

Interpersonal interaction is ubiquitous and plays a critical role in various contexts. When individuals interact with each other, they form emotional bonds and establish affective connections. These interactions usually occur through nonphysical forms, such as eye contact, verbal communication, and facial expression, or physical interactions, including touch [3]. Even the copresence of strangers who pay joint attention to a movie or a concert has been shown to establish social connections, leading to the creation of interpersonal interactions [4]. During interpersonal interactions, paraventricular nucleus oxytocin neurons exhibit heightened activity, which promotes prosocial behavior by providing excitatory input to reward-specific dopamine neurons in the ventral tegmental area [5]. This mechanism fosters rewarding experiences in social contexts, enhancing the benefits of interpersonal exchanges. Furthermore, individuals with social behavioral deficits, such as those seen in autism spectrum disorder (ASD), can derive marked benefits from this oxytocin-driven process [6]. Beyond its social effects, the activation of dopamine neurons contributes to positive emotions [7] and plays a key role in motor skill learning as well as motor memory consolidation [8], which also provides a potential avenue for enhancing the effectiveness of functional robots, such as rehabilitation systems.

A harmonious interpersonal relationship generates prosocial behavior and is beneficial for establishing rapport. Furthermore, it contributes to greater pleasurable emotional experiences [9], improved neurodevelopment [10], increased self-efficacy [11], more participation [12], successful cooperation, and better performance [13]. In the context of clinical therapy and healthcare, effective interpersonal interaction could also lead to a positive patient–clinician relationship, which reflects positive affectivity [14] and induces greater patient satisfaction, mutual trust [15], treatment adherence [16], and superior clinical outcomes [17]. Research on placebo effects has shown that, in some cases, altering the interpersonal component can lead to substantial improvements in symptoms, behavior, and even underlying brain processes [18,19].

The value of interpersonal interaction in healthcare lies primarily in its capacity to evoke empathic processes. Empathy is widely recognized in psychology as a multidimensional construct. Bloom [20] identifies 4 primary forms of empathy: (a) cognitive or intellectual empathy, the conceptual understanding of another person’s emotional state without necessarily sharing it; (b) emotional contagion, the spontaneous transmission of affect between individuals; (c) affective empathy, the experience of emotions inferred from others; and (d) compassion, concern for others’ well-being accompanied by a motivation to alleviate their suffering. These distinctions underscore not only the complexity of human empathy but also the inherent challenges in replicating its processes in the absence of direct interpersonal engagement.

However, healthcare systems are increasingly facing severe resource constraints, making it difficult to ensure consistent and high-quality empathic interactions between patients and clinicians. To address this deficit, emerging technologies have begun to replicate aspects of empathy through computational models. In this context, artificial empathy refers to the ability of machines to perceive, interpret, and simulate empathic responses during human–machine interaction. Rather than reproducing genuine affective experience, artificial empathy operates through algorithmic recognition and response, offering a functional approximation of empathic interaction. Artificial empathy, therefore, seeks to translate the benefits of human empathy into computationally mediated forms of care.

This review summarizes 3 major technological platforms: multiplayer games, social robots, and virtual agents. They represent distinct but complementary pathways to embedding artificial empathy into therapy and healthcare. We highlight how these systems incorporate interpersonal interaction and trace their progression toward increasingly advanced methods designed to replicate complex human dynamics and achieve artificial empathy.

## Technical Platforms

**Fig. 1.**
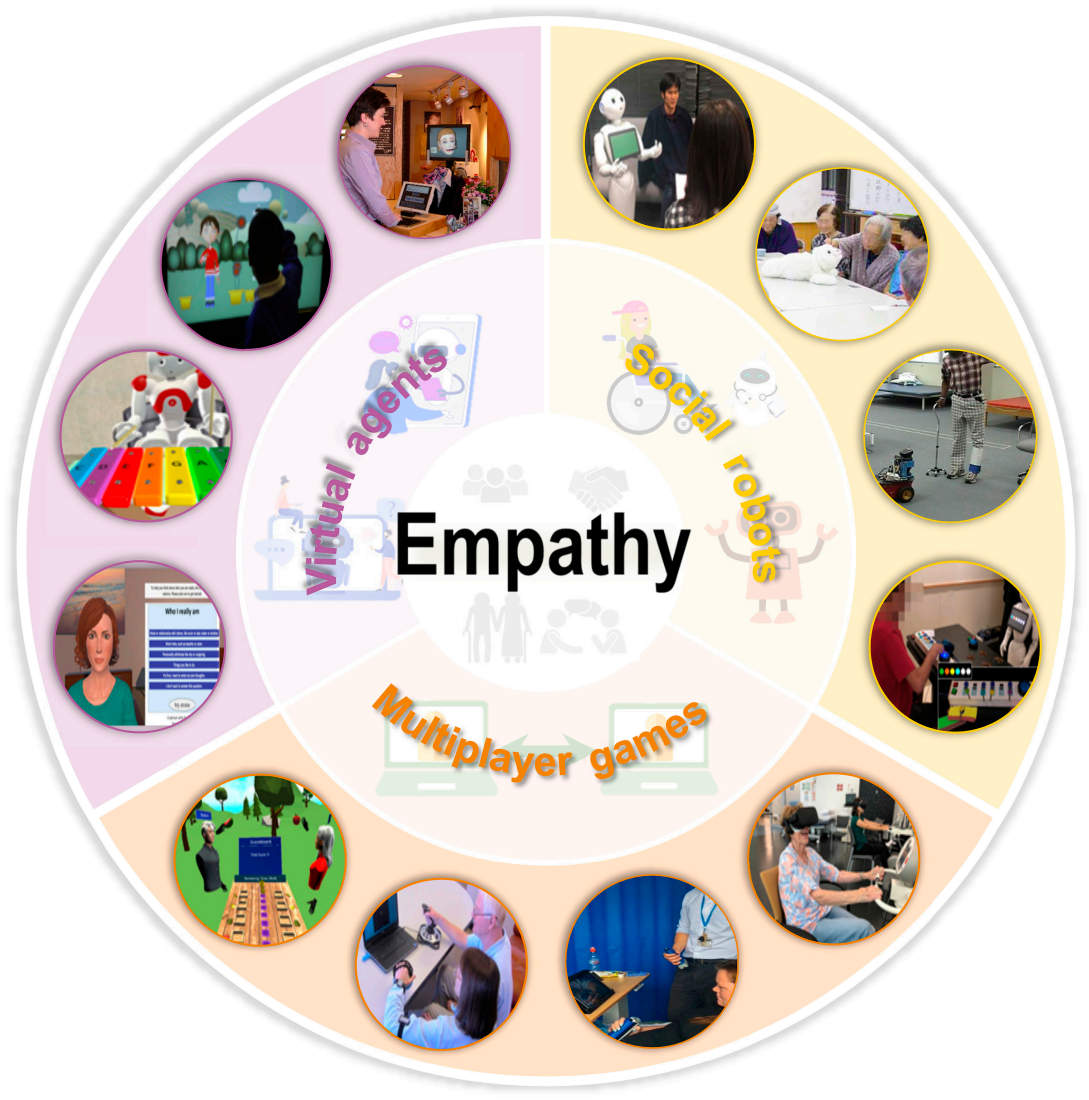
Techniques enhancing empathy or artificial empathy. This review highlights 3 approaches that foster empathy or artificial empathy through mimicking interpersonal interaction: multiplayer games that strengthen real-time social connections, and social robots or virtual agents that simulate human-like interactions.

**Fig. 2.**
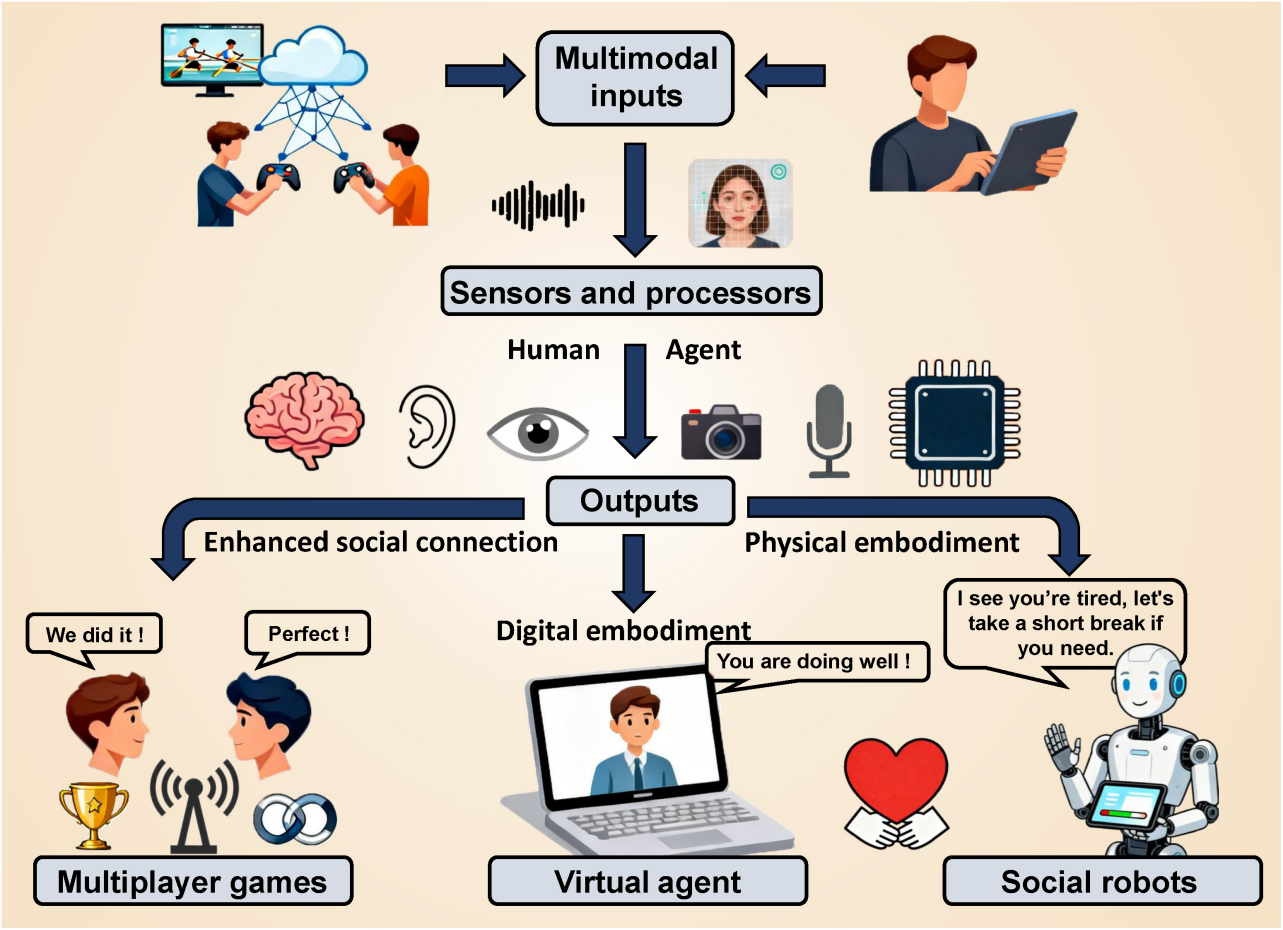
A general pipeline for eliciting artificial empathy throughout technical platforms. Multimodal signals are collected for both human and agent processing. Human perception occurs through sensory organs with brain computation, while the agent utilizes sensors and processors. Generated responses form a real-time closed-loop interaction, eliciting artificial empathy throughout the process.

Interpersonal interactions manifest in various forms, including cooperation, competition, praise, encouragement, and comfort. These interactions can be facilitated through several technical platforms. As shown in Fig. 1, we categorize these into 3 areas: (a) multiplayer games, which provide platforms for real-time interpersonal interaction technologies that replicate human–human social interactions; (b) socially embodied robots that enable direct physical engagement; and (c) virtual agents that utilize computer screens and advanced digital interfaces as mediums for interaction. Fig. 2 illustrates a general pipeline for eliciting artificial empathy throughout the aforementioned 3 types of technological platforms. Multimodal signals from the environment are collected and input to both humans and agents. For humans, sensory organs such as eyes, ears, and tactile receptors function as sensors to gather information, which is then processed by the brain. For agents, inputs are collected via devices and processed by processors, such as cameras, microphones, and flexible sensors [21,22]. Responses are subsequently generated based on decoding results. This real-time closed-loop pipeline enables interpersonal and human–agent interaction, and artificial empathy will be elicited throughout this process.

### Multiplayer games

Rehabilitation therapies integrated with digital games have enhanced patient engagement and therapeutic outcomes [23]. While considerable research has focused on making games more appealing to motivate patients during rehabilitation [24], less attention has been given to the social relevance of these games. Individuals can derive enjoyment from social interactions and make more effort when playing multiplayer games. Multiplayer games provide effective platforms for facilitating interpersonal interactions both between patients and between therapists and patients [25].

The technical implementation of games in healthcare was first envisioned for patient–therapist collaboration in telerehabilitation [26]. Later, it was observed that under the telerehabilitation system, individuals preferred to perform rehabilitation tasks with a human rather than a computer, which is similar to their preference for playing general computer games [27]. Consequently, multiplayer games are expected to motivate patients and enhance the potential of rehabilitation therapy, such as robot-assisted therapy [28]. Table 1 summarizes some typical multiplayer games designed for rehabilitation. Multiplayer modalities have been well combined with various digital games in recent years. Several studies leveraged the multisensory feedback capabilities of virtual reality (VR) [29] and introduced multiplayer modes, thereby offering higher-quality and more immersive interpersonal interactions [30]. Effective integration of multiplayer games with motor rehabilitation devices has been achieved for both upper and lower extremity training [30,31]. Unlike robots or virtual agents, multiplayer games do not simulate empathy through algorithmic design. Instead, they function as a mediating technology that extends genuine human-to-human empathy into digital environments. This makes them a hybrid case within the framework of artificial empathy. Although empathic processes are inherently human, the technological platform enables their manifestation in remote or resource-constrained therapeutic contexts.

**Table 1.** Multiplayer games for therapy and exercise

Authors and references	Players’ type	Rehabilitation device	Mode	Game task	Game platform	Subjects	Measures
Ribeiro et al. [32]	Patient–patient	Not used (communication skill training)	Cooperation, single-player	Communication with each other	Tablet	4 children diagnosed with autism	Game performance
Novak et al. [31]	Patient–patient	ARMin robot (rehabilitation robot for upper extremity)	Competition, cooperation	Air hockey (simulated batting task)	Computer	8 impaired chronic stroke subjects	(a) Intrinsic Motivation Inventory (IMI); (b) game performance; (c) personality questionnaire
Gorsic and Novak [115]	Patient–friend/family	BiMeo arm rehabilitation system	Competition, cooperation, single player	Variants of the classic Pong game	Computer	5 participants with neurological injuries and 2 participants with orthopedic injuries	(a) IMI; (b) game experience questionnaire; (c) personality questionnaire; (d) exercise intensity
Gorsic et al. [116]	Patient–friend/family, patient–patient	BiMeo arm rehabilitation system	Competition, single player	Variants of the classic Pong game	Computer	15 participants with chronic arm impairment and 20 in the acute or subacute stroke phase	(a) IMI; (b) game experience questionnaire; (c) personality questionnaire; (d) exercise intensity
Mace et al. [117]	Patient–expert	Handle (participants apply grip force to it)	Cooperation, single-player	Balloon balancing task	Computer	100 stroke patients with arm paresis secondary to acute stroke	(a) Game performance; (b) engagement and user experience questionnaire
Pereira et al. [40]	Patient–patient	Handle (exercise for upper extremity)	Competition, coaction, collaboration	Catching balls	Computer	20 pairs of community-dwelling older adults	Engagement questionnaire
Catalán et al. [23]	Patient–patient	Rubidium device (rehabilitation robot for upper extremity)	Cooperation, single player	Apple-picking (point-to-point movement task)	Computer	14 stroke patients	(a) Exercise intensity; (b) physiological response: galvanic skin response (GSR), heart rate (HR); (c) questionnaire: patients’ preferred game mode
Shah et al. [118]	Patient–patient	Not used (voluntarily full-body exercise)	Cooperation, single player	Fruit placement	Virtual reality	14 elderly people who had experienced some reduction in their physical strength due to physical inactivity	(a) IMI; (b) physical exertion
Høeg et al. [30]	Patient–friend/family	Motomed Viva 2 (rehabilitative recumbent bike for lower extremity)	Cooperation	Virtual tandem bike	Virtual reality	11 stroke patients and their coplayer	(a) IMI; (b) interpersonal Interaction (IPI) questionnaire
Baur et al. [119]	Patient–patient	Armeo Spring	Competition, cooperation, single player	Air hockey game	Computer	20 stroke patients	(a) Game performance; (b) IMI
Shih et al. [120]	Patient–therapist	Not used	Cooperation	Multiplayer interaction training	Virtual reality	20 stroke patients	Questionnaire

Research presented in Table 1 demonstrated that patients exhibited superior performance in multiplayer mode compared to single-player mode. The most commonly used measures are subjective experience questionnaires. On the other hand, researchers have also suggested using physiological responses such as galvanic skin response (GSR), heart rate (HR), and electromyogram (EMG) to assess patients’ performance more quantitatively and objectively [32]. Nevertheless, evidence shows that the benefits of multiplayer modes are not universal. Some studies reported no significant clinical gains or even stress responses, while others found that positive effects on social involvement did not consistently translate into motor recovery [33,34]. However, the multiplayer component is rarely addressed in therapeutic games designed for individuals with mental illness [35]. For instance, it has been suggested that computer games could potentially exacerbate the isolation of individuals with autism rather than foster a collaborative environment [36]. On the other hand, the incorporation of multiplayer elements into therapeutic games for autism has been widely advocated, emphasizing their potential benefits [32]. In the context of older adults with dementia, no significant increase in social interaction was observed, even when social gaming contexts were introduced [37]. Despite this, researchers continue to recommend further exploration of strategies to enhance engagement and interaction among people with dementia through therapy games [37].

Furthermore, different interaction modes may have varying impacts on players. Multiplayer games provide different modes to specify the players’ interaction type. The most common modes are competition and cooperation [25,31], which have been demonstrated to increase physical performance and motivation in rehabilitation therapies [25]. Competitive gameplay may lead to more intensive exercise [31]. Subjects who enjoyed competition reported greater effort in the competitive mode [31]. It has been demonstrated that the competitive game mode is comparable to the high-difficulty single-player game mode in terms of difficulty level and intensity [23]. Meanwhile, some studies argue that competitive games may decrease intrinsic motivation [38], lead to patients’ stress [25], and increase aggressive behavior. A limited evaluation indicated that competitive games often made patients feel discouraged and awkward [39]. These conflicting results suggest that the effects of competitive modes are highly context-dependent. Individual factors such as patients’ tolerance for stress, enjoyment of competition, and psychological resilience may shape outcomes. Consequently, competitive gameplay should not be considered universally beneficial, but rather as an approach that requires careful tailoring to patient characteristics and therapeutic goals. It was suggested that cooperative games may be a safer choice for rehabilitation [31]. In addition, some studies also addressed other interaction modes, such as collaborative, which is easily confused with cooperative. Both cooperation and collaboration mean working together to complete tasks, but collaboration also implies that the players have the same role [40]. It has been observed that collaboration elicited higher social involvement in empathy, positive affect, and behavioral engagement [40]. However, the consensus among most researchers is that the suitable game mode for subjects strongly depends on each individual as well as their coplayer [41] and the choice of rehabilitation games should be made carefully, according to each patient’s personality.

In summary, multiplayer gaming is a valuable methodology for constructing social connections between users, even when they are geographically separated. People can choose interactive games based on their preferences, opting for either competitive or cooperative options. By participating in multiplayer games, users can benefit from interpersonal interaction and collaborative engagement. Although real-life interpersonal interactions offer the most direct form of social support for rehabilitation, pairing patients with suitable partners is not always feasible, partly due to the limited availability of healthcare personnel. Patients often face difficulties in finding a suitable partner when they wish to begin game-based rehabilitation or other training. Exploring techniques to simulate human-like interactions offers a promising solution to address this challenge.

### Social robots

Social robots can interact with humans through touch, gaze, gestures, facial expressions, and speech. Leveraging social cues in human–robot interaction has been shown to modulate human neural activity [42] and provides an engaging, effective complement to traditional human-to-human interaction. Such robots are designed to support natural, reciprocal exchanges with people [43] and are expected to be deployed across healthcare, domestic, educational, and workplace settings. In healthcare and therapeutic settings, social robots have shown considerable potential to assist with activities of daily living, guide physical exercise, and encourage emotional expression. They provide a promising complement to human care by supporting recovery and improving healthcare outcomes, while maintaining patient motivation and engagement throughout therapy [44]. Table 2 summarizes representative humanoid robots developed for rehabilitation, exercise assistance, and daily support. These systems have generally been well received by users, as reflected in positive evaluations and task performance outcomes.

**Table 2.** Humanoid social robots for therapy and exercise

Authors and references	Robot name	Interaction type	Task	Subjects	Measures	Results/Conclusions
Matarić et al. [44]	Pioneer mobile robot	Conversation	Monitor movements of stroke patients, provide reminders, prompt, praise, and encouragement to the patient	6 stroke patients	Questionnaire about the robot is well-received or not	Robots are positively received by stroke survivors and encourage their commitment to prescribed rehabilitation exercises
Kidd et al. [51]	Autom	Eye contact, gestures, conversation	Help an individual track information related to their weight loss program.	45 people trying to lose and keep off weight (17 to 72 years old)	Questionnaire about the length of time participants use the system and their relationship with the system	Participants tracked their calorie consumption and exercise longer when using the robot and developed a closer relationship with the robot
Sabelli et al. [121]	Robotive	Remotely controlled dialogues and child-like behaviors	Communicate with the elderly and support the elderly center staff	55 elderly people (average age, 83.9 years)	Interviews and observations about interactions between the elderly and the robot	The robot was well received due to its “child” role and behaviors like greeting users and addressing them by name
Wainer et al. [122]	Kaspar	Gestures, facial expressions, and speech (prerecorded messages)	Engage, motivate, encourage, and advise pairs of children playing an imitation game	6 children with autism spectrum disorder (ASD)	Video of the children’s play sessions	Children with ASD exhibited enhanced social behaviors when interacting with peers after paired play with the robot compared to before
Valadão et al. [46]	MARIA	Eye gaze, touch	Interact with the children and stimulate their social skills, such as eye gaze, touch, and imitation	10 children (50% with ASD)	(a) Children’s reactions measured by video recording; (b) questionnaire about behaviors of the children toward the robot	ASD children interacted with the robot more and improved their social skills
Feingold-Polak et al. [49]	Pepper	Friendly greetings	Give the patients instructions and feedback on their performance, and track their performance	24 post-stroke patients	Questionnaires about users’ satisfaction and opinion on the system in detail, and technical limitations	Patients exhibited greater acceptance of the robot, which proves beneficial for long-term rehabilitation of post-stroke patients in a clinical setting
Marino et al. [47]	NAO	Body movements, verbal phrases, nonverbal gestures, face tracking for gaze maintenance	Reward, encourage, engage children’s attention and motivation while delivering positive reinforcement	14 children with ASD	(a) Direct observations of the children; (b) emotional comprehension and lexicon test	Social robots enhance children’s understanding of emotions within specific social contexts
Céspedes et al. [50]	NAO	Verbal phrases, nonverbal gestures, face tracking for gaze maintenance	Motivate, monitor performance, and give feedback	20 patients during cardiac rehabilitation phase	(a) Dropout rate; (b) physiological parameters; (c) interaction with the robot; (d) questionnaire about the patients’ and clinicians’ perception of robot	The dropout rate was lower in the robot-assisted condition, with greater improvements in cardiovascular function and recovery. Patients consistently engaged with the robot throughout long-term rehabilitation. Both clinicians and patients gave positive feedback on the robot
Lee et al. [123]	NAO	Verbal instructions, visualization, and gestures	Monitor motor training, and give corrective feedback	15 post-stroke, and 10 healthy participants	(a) User experience questionnaire; (b) model’s performance in identifying movements that need correction	Users showed positive feedback. The system can accurately assess users’ movement quality and adapt to new users

A major application of social robotics lies in supporting individuals with ASD [45]. Recent studies demonstrated that ASD children responded more favorably to robots, whose predictable and consistent behavior contrasted with the inherent variability of human interactions [46]. Because initiating social engagement is frequently challenging for individuals with ASD, robot-assisted interventions offer a promising means of fostering cognitive and social development. For example, Marino et al. [47] randomly assigned 14 children to a group-based cognitive behavioral program, either incorporating or excluding a robotic assistant, and found significant improvements in the children’s ability to recognize, understand, and interpret emotions when the robot was present. Additional studies have reported that robot-mediated therapy reduced stress, improved communication, and helped regulate impulsive behavior in individuals with ASD [48].

Another important application of social robots in the medical field is physical rehabilitation and exercise. Unlike a rehabilitation robot, which directly manipulates or assists the patient’s limbs to perform specific actions, the social robot typically serves as a coach or guide, providing psychological support to the user. The repetitive and prolonged nature of rehabilitation and exercise programs often induces negative emotional states that hinder patient adherence. Social robots can help sustain engagement by monitoring progress in therapy and daily activities while providing consistent encouragement and personalized feedback [44]. Studies have demonstrated their benefits for individuals recovering from stroke or heart disease [49,50], who showed high levels of acceptance and greater willingness to adhere to rehabilitation protocols. Similarly, participants in a weight-loss study exercised for longer durations and reported stronger emotional connections when assisted by a robot coach [51]. In daily care, social robots have also proven effective in promoting both physical and psychological well-being among older adults. Wada and Shibata [52] introduced robotic companions into a residential care facility, where residents interacted with them for more than 9 h per day. After 1 month, participants exhibited increased communication not only with the robots but also with one another, alongside measurable reductions in physiological stress markers and improvements in emotional well-being—effects that may help mitigate the risks of illness and cognitive decline.

**Fig. 3.**
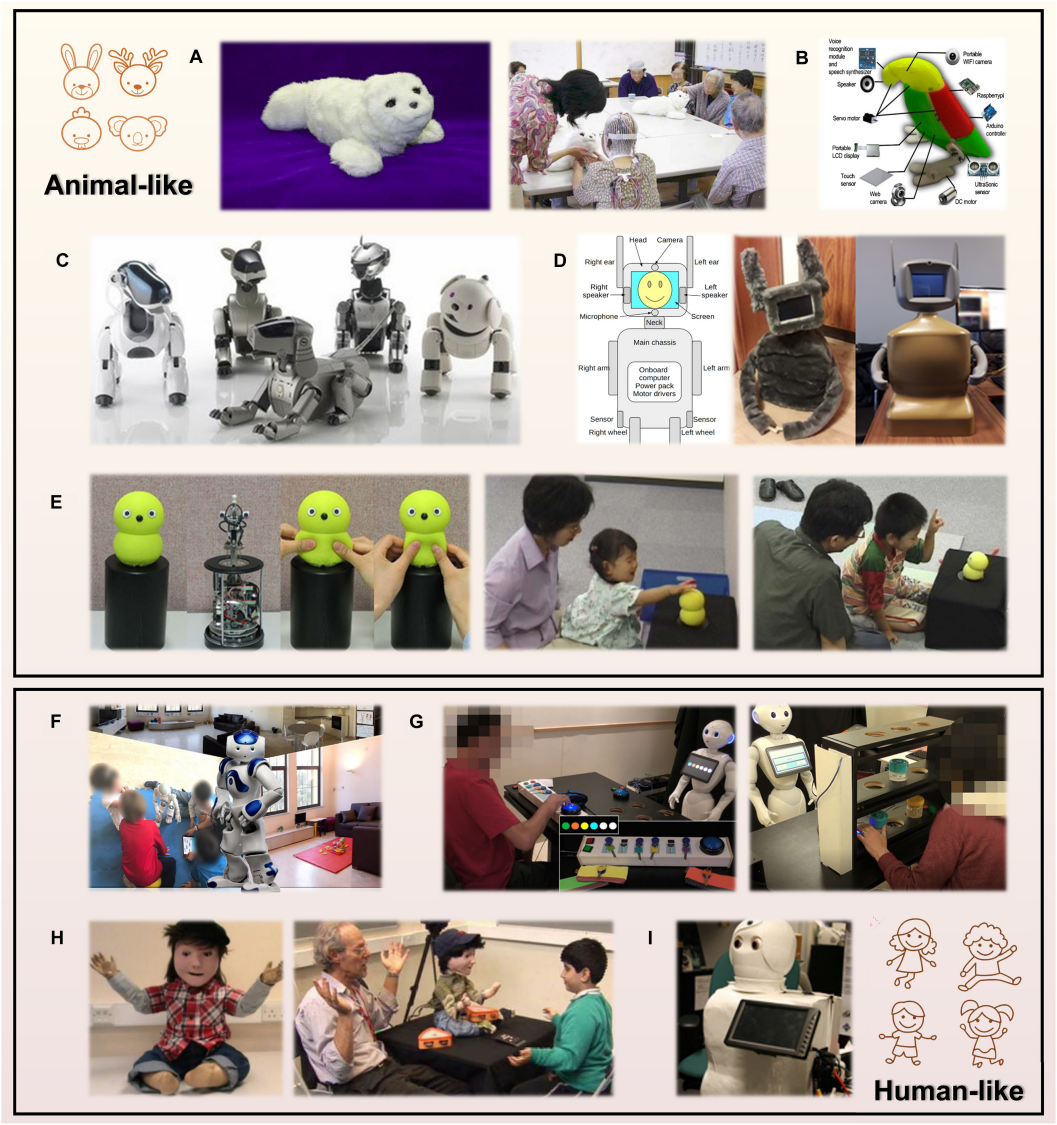
Social robots with zoomorphic (animal-like) and anthropomorphic (human-like) appearances in various scenes of healthcare. (A) Paro and its application in the nursing home [61]. (B) KiliRo robot [48]. (C) AIBO [113]. (D) DreamRobot [64]. (E) Keepon and its application in autism spectrum disorder (ASD) therapy [63]. (F) Nao [47]. (G) Pepper and its application in stroke therapy [49]. (H) Kaspar and its application in ASD therapy [114]. (I) Autom robot [51].

Table 2 shows that social robots are equipped with diverse configurations and functions, providing a solid foundation for enriching their interaction types. These robots have the potential to initiate interpersonal interactions through various means, such as appearance, behavior, speech, and gestures. For instance, NAO and Pepper, 2 typical humanoid robots, are equipped with 20 motors in their arms and legs, allowing them to perform a wide range of movements and gestures with high flexibility [53]. The embodiment of robots in physical form is believed to have a substantial impact on their functionality and effectiveness [54]. Among various factors, a robot’s physical appearance seems to have the most important influence on shaping people’s initial perceptions and expectations. For example, Lohse et al. [55] concluded from an online survey that participants regarded the robot’s appearance as more important than its function. Likewise, DiSalvo et al. [56] found that the facial features of robots influenced their perceived human likeness. For these reasons, many social robots are designed with an anthropomorphic appearance. However, as suggested by Mori’s Uncanny Valley theory [57], the excessive human-like appearance of robots is not always well-accepted. In addition, Dautenhahn [58] argued that anthropomorphism could create unrealistic expectations about a robot’s cognitive and social capabilities. Users might expect natural interactions and authentic emotional expressions, rather than preprogrammed responses or mechanical gestures [59]. When humanoid robots fail to meet these expectations, users may become disengaged or even cease interactions altogether [60]. This issue is particularly likely to emerge over time, potentially hindering the sustainability of long-term human–robot interaction [59]. To address this issue, researchers have suggested that zoomorphic or cartoon-style robots may provide a better solution (Fig. 3), as individuals tend to expect less from a robot with an animal or cartoon appearance. Furthermore, robots designed to resemble appealing animals or cartoon characters are generally less likely to evoke fear or discomfort in users. Various animal-shaped robots have been successfully implemented in healthcare, including Paro (seal-like) [52,61], AIBO (dog-like) [62], KiliRo (parrot-like) [48], Keepon (chicken-like) [63], and DreamRobot (rabbit-like) [64]. These robots with a charming appearance have been commercialized and are widely accepted by users in healthcare settings such as hospitals and nursing homes. Nevertheless, more integration of human-like characteristics could enhance the functionality of social robots, though navigating the Uncanny Valley remains a significant challenge. As technology advances, social robots are likely to exhibit increasingly fluid and natural human-like behaviors, positioning them as an integral part of daily life. Over time, the general public may become accustomed to robots with highly realistic human features, reducing the likelihood of fear or disappointment [65]. Currently, many scholars remain engaged in this endeavor and have already attained noteworthy outcomes. For instance, instead of relying on preset or teleoperated speeches or responses, Bertacchini et al. [66] innovatively connected the social robot Pepper with the large language model (LLM) ChatGPT to facilitate real-time dialogue generation. The incorporation of artificial intelligence (AI) generation technology markedly enhances the robots’ autonomy, flexibility, personalization, and independence, making their responsiveness more appealing to users. For example, ChatGPT provides higher-quality and more empathetic responses to patients’ healthcare inquiries, with users preferring its answers over those of physicians [67]. To enable facial coexpression during human–robot interaction, Hu et al. [68] proposed a deep learning framework that predicts humans’ facial expressions, allowing the robot to smile simultaneously. Although limitations in accuracy, processing speed, and resource availability remain and may affect the overall performance of human–robot interactions [66], the integration of AI generation technologies has undeniably brought significant advancements to social robots.

### Virtual agents

**Fig. 4.**
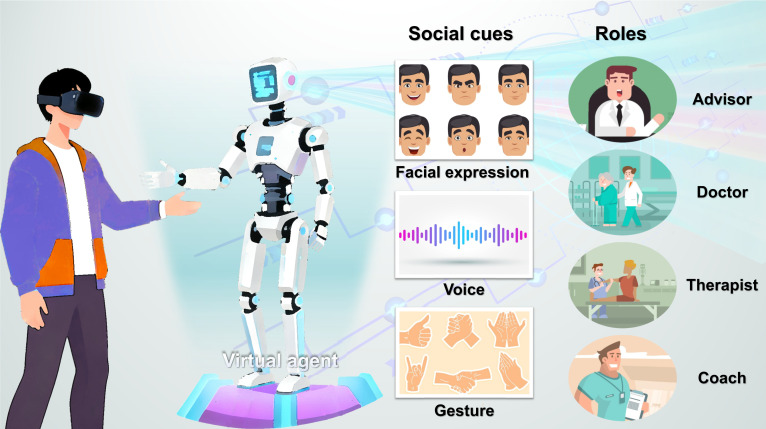
Virtual agent with nonphysical embodiment. It exists in the digital world but could be embodied in various roles and interact with users through a range of social cues.

The use of virtual agents with nonphysical embodiment may represent an effective approach to social interaction with users without the issue of high costs typical of social robots. Conventional virtual agents are capable of interacting with users through the simulation of human characteristics via computer interfaces. It can be presented to the user with a virtual appearance or as a chatbot that exclusively communicates through text [69]. As shown in Table 3, virtual agents can play many similar roles to social robots in the medical field, such as companions, advisors, coaches, therapists, or doctors. Except for physical touch, as shown in Fig. 4, they are capable of simulating multiple forms of interpersonal interaction, including facial expressions, gestures, verbal communication, and eye contact.

**Table 3.** Virtual agent with nonphysical embodiment for therapy and exercise

Authors and reference	Platform	Interaction type	Task	Subjects	Measures	Results/Conclusions
Bickmore et al. [124]	Computer	Communication	Act as an exercise advisor and interact with subjects daily	10 elderly participants	(a) Interaction history; (b) step count; (c) questionnaires; (d) semi-structured interview	Most subjects highly rated the agent as a trusted friend and desired post-study interaction
Anderson-Hanley et al. [125]	Virtual reality	Competition	Competitive and stimulate exercise experience	14 independent living older adults	(a) Pedaling effort (watts); (b) participants’ self-reported competitiveness index	The addition of a competitive avatar did not negatively affect less competitive riders but boosted the exercise effort of more competitive riders
Bernardini et al. [126]	Multitouch LCD display	Communication, behavior, gaze, gesture, facial expression	Acts as a peer and tutor to practice social communication skills	29 children with ASD and/or other disabilities	Video of child’s interaction behavior (responds and initiations to the interaction)	Some children benefited from their exposure to the agent and the overall ECHOES environment
So et al. [127]	Computer	Behavior, verbal phrases	Teach gestural comprehension and production	20 children with ASD	(a) Children’s visual-motor coordination skills; (b) neuropsychological test; (c) children’s learning outcomes	Children applied gestures learned from a robot to a human researcher, confirming the robot animation’s teaching effectiveness
Abdullah et al. [128]	Tablet	Communication	Act as a professional smoking cessation counselor to encourage participants to stick with their quitting plan	6 smoking veterans	(a) Agent’s usefulness and usability; (b) participants’ satisfaction and likelihood to recommend; (c) participants’ smoking behavior	The virtual agent was endorsed by all participants as a useful tool for them to set a quit date, with high satisfaction and recommendation rates
Shahab et al. [81]	Virtual reality	Behavior, verbal phrases	Guide and teach musical skills	12 children with ASD	(a) Video record of children’s performance; (b) the kinematic data of the headsets and controllers; (c) assessments by psychologists; (d) questionnaires	The tech improved musical phrasing in children with ASD, but cognitive gains were limited by a ceiling effect
Richards et al. [70]	Computer	Communication	Take on the responsibility of stroke patients’ rehabilitation and the process of re-establishing themselves as a person independent of the effects of the stroke	8 stroke patients	(a) Full digital session record; (b) questionnaire about patients’ feedback	The virtual agent was generally well accepted and considered useful
Sestino and D’Angelo [129]	Computer	Communication	Remote teleconsultation in an online environment	689 international participants	Questionnaire about users’ feedback	Perceived anthropomorphism is positively associated with the intention to use digital healthcare services
Dar et al. [130]	Computer	Behavior, verbal phrases	Breathing exercises guided by a virtual coach equipped with a human appearance, voice, and simulated breathing physiology	20 healthy participants	(a) Questionnaire about users’ feedback; (b) breathing task performance	The coach’s instructional influence showed no significant difference across task difficulty levels. Participants were open to replacing a human coach with a virtual one

While several studies have highlighted user preferences for physically embodied robots over virtual avatars, it remains essential to optimize the interactive capabilities and broaden the applications of virtual agents [49]. In certain contexts, virtual agents have even outperformed their embodied counterparts [70]. The absence of physical embodiment also provides greater flexibility in deployment, removing many of the spatial and logistical constraints that limit robotic systems. Moreover, virtual agents are inherently suited to digital environments and can be seamlessly integrated into therapeutic games, exercise platforms, and daily-care applications. To address the inability of virtual agents to engage in physical interaction within virtual environments, haptic interfaces have been widely explored and applied in VR to enhance users’ immersive experiences [71]. In digital settings, haptics facilitated by devices such as handheld controllers [72] and wearables [73] can simulate physical interaction through real-time tactile feedback. To liberate users from handheld devices and enable more natural interaction, mid-air haptic technologies have been proposed, which utilize ultrasonic transducers to generate tactile sensations via sound waves [74].

Virtual agents primarily communicate with users through visual and auditory modalities, with their appearances typically rendered in animated form. Over the past decades, advances in digital modeling have enabled increasingly expressive representations of such agents. From computer-generated 2-dimensional (2D) and 3-dimensional (3D) rendering to holography [75,76], VR [77], augmented reality (AR) [78], mixed reality [79], and extended reality [80], digital visualization technologies have continued to integrate and evolve. VR can deliver a fully immersive, multisensory interactive experience, and several studies have demonstrated its effectiveness in simulating interpersonal interactions with patients. For example, Shahab et al. [81] created a virtual music classroom and a teaching robot on a VR platform for children with autism. After a period of training, participants showed modest improvements in cognitive ability and a general upward trend in musical performance. AR serves as a novel medium that enriches visual content by superimposing virtual elements onto the physical environment in real time [82], offering a complementary approach to VR. AR is frequently associated with holographic technology to generate highly realistic 3D imagery [83]. Although AR-based interpersonal interaction has not yet achieved widespread application compared to VR, emerging applications are beginning to appear [75,78].

Continued advances in immersive technologies are gradually blurring the boundary between the physical and digital worlds. Increasingly sophisticated digital modeling enables highly anthropomorphic avatars with vivid facial expressions and body movements, thereby deepening users’ sense of presence and engagement in social contexts. A multitude of behavioral models and animation engines have been developed to assist avatars in selecting appropriate verbal and nonverbal behaviors according to the interaction context [84,85]. However, these systems remain constrained by computational limits and rule-based architectures, leading to preprogrammed, rigid behavior that often feels mechanical or unresponsive to nuanced human cues. Their inability to accommodate individual differences, contextual variability, and emotional subtleties continues to impede the development of natural and adaptive human–machine interaction. The integration of AI offers an effective way to overcome the current limitations. AI technology enables virtual agents to fuse multimodal inputs, perform context-aware decision-making, and generate adaptive responses tailored to user states. LLM, as one of the core techniques in AI generation, has already demonstrated strong capabilities in context understanding, decision reasoning, and the generation of fluent, human-like dialogue and behavior during interaction [66,86]. Generative AI (GenAI) models further extend this capacity to realistic digital content creation, including photorealistic avatars, voice synthesis, and expressive facial animation features that enhance realism and user engagement. Pataranutaporn et al. [87] developed a pipeline to create humanoid characters with facial expressions, voices, and movements using advanced multimodal conversion techniques (text-to-audio, audio-to-video, and video-to-video). The convergence of AI-driven character generation with virtual agents holds the promise of facilitating enhanced personalization and fostering greater trust [87], supporting their adoption in healthcare and therapeutic settings. Furthermore, merging AI with immersive platforms such as VR and AR is expected to drive the next major breakthrough in virtual-agent technology, bringing genuinely adaptive, emotionally intelligent digital companions into clinical practice.

## Toward Enhanced Artificial Empathy

Robots and virtual agents have been widely adopted not only in the medical field but also in education, service industries, and other sectors. The integration of AI technologies and immersive media, such as VR and AR, is particularly effective at making agents’ decision-making and action processes more human-like. These technologies have made notable strides in anthropomorphizing, enabling agents to exhibit human-like expressions, gestures, and conversational dynamics. Enhancing such human-like interaction fosters social connections between users and agents, thereby improving acceptance and satisfaction. Nonetheless, these preliminary imitations remain far from replacing, let alone surpassing human interaction. Future work should focus on amplifying the positive effects of interpersonal engagement through technological innovation, particularly those linked to emotional and cognitive resonance. For example, in educational contexts, synchronized neural activity has been observed between teachers and students during active classroom engagement [88]. Similarly, in clinical therapy, Ellingsen et al. [89] found that brain-to-brain concordance mediated analgesic outcomes in therapy; such synchrony underpins therapeutic alliance and positive affect, leading to improved clinical results. However, these interactional benefits have yet to be fully replicated by current robotic and virtual systems.

To address this limitation, researchers must develop artificial empathy that can perceive, understand, and respond to users’ physiological and psychological needs. Current approaches primarily aim to interpret human cognitive and emotional states while generating responses that simulate empathy, thereby eliciting user trust and engagement. With the continuous advancement of sensing technologies, the collection and monitoring of users’ multimodal information during interactions have become increasingly accessible, while powerful computational systems enable the integration and interpretation of vast information streams. These capabilities enable the precise estimation of users’ states, potentially exceeding human perceptual accuracy in certain tasks. Many agents can already detect and monitor users’ physical conditions [50], and integrating cognitive and affective recognition remains essential for a comprehensive understanding of human behavior. Researchers are therefore exploring ways to infer users’ psychological and cognitive states in real time.

A crucial aspect of this effort is the ability of agents to “understand” human emotions. There is an increasing expectation for agents to perceive, understand, and express emotions at a level comparable to humans. Emotion recognition has thus emerged as a central focus, offering an objective method to automatically capture human emotions, in contrast to traditional subjective questionnaires and scale assessments. This capability not only facilitates affective interaction between agents and humans but also holds particular significance in the medical field. Patients experience greater therapeutic benefit when perceiving empathic understanding from an agent, though some scholars argue that this represents a form of pseudo-empathy [90]. For patients with psychological disorders, real-time emotion recognition can function as a mental health monitor, supporting the maintenance of psychological well-being [91].

Methodologies for automatic emotion recognition commonly rely on behavioral cues such as voice, text, facial expressions, body gestures, and eye tracking. For instance, Kashii et al. [53] utilized the humanoid robot Pepper to improve interactions with patients with motor disabilities by recognizing emotions from video and speech. In parallel, physiological signals, including electroencephalogram (EEG), electrocardiogram (ECG), EMG, HR, skin temperature, respiration rate, and GSR, have also been employed to infer emotional states [92]. These physiological indices are often preferred over behavioral indicators, as they provide a more reliable approach to mitigating social desirability bias and reducing the potential for deception. Machine learning remains a dominant approach in this field. It applies to assess users’ emotional dynamics and create closed-loop systems for emotional regulation. OPO-FCM [93] and emoLDAnet [94] were successively proposed to use facial expressions for emotion decoding, demonstrating efficacy in identifying emotional states, particularly depression and anxiety. A dual-branch joint network was developed to improve cross-subject emotion recognition [95]. Snoek et al. proposed a prediction–explanation–exploration framework to quantify the performance of different models in predicting human emotion based on facial expressions [96]. Tan et al. [97] proposed a Monte Carlo-based multimodal emotion recognition method that combines emotion classification results from facial expressions and EEG to optimize human–robot interaction. Du et al. [98] developed a social robot with an integrated emotion recognition and feedback model for elderly people, combining voice, semantic, and topic-based emotion recognition with convolutional neural networks and long short-term memory algorithms. This model refines emotional information from audio and text data, with a feedback module that generates appropriate facial expressions based on the recognized emotions.

The field of emotion recognition is now trending toward contextual approaches that incorporate attention mechanisms, transformers, and particularly graph-based techniques. Advances in hardware, such as monolithic synaptic devices for affective computing [99], further support real-time emotion decoding. The integration of multimodal data, advanced hardware, and algorithms has greatly enhanced emotion recognition, paving the way for more effective and empathetic human–agent interactions, especially in healthcare and psychological support. However, much of the existing work prioritizes classification accuracy, while overlooking the need for ecological validity in real-world settings. Many models still rely on public datasets where emotions are elicited through images or videos, which differ greatly from genuine clinical scenarios [100]. Moreover, cultural differences in how emotions are expressed and perceived further complicate recognition, as models trained on data from one population may not generalize well to others. Sustained effort is required to ensure reliable emotion recognition across diverse clinical environments. In addition to emotion recognition, other psychological and cognitive metrics, such as trust [101], engagement [102], the agent’s social presence [7], and human–agent rapport [103], are also conducive to the interaction. For example, SCIANN was developed to decode interpersonal empathy during human cooperative tasks using EEG [104], which also holds promise for assessing whether artificial empathy is elicited during human–robot interactions. However, despite ongoing research, there is still a notable absence of accurate, real-time, and quantitative models for evaluating these metrics. Moreover, enabling agents to flexibly adjust their behavior in response to real-time fluctuations in these metrics remains a significant challenge.

Beyond real-time adaptations, emerging research increasingly highlights the significance of tailoring interaction styles to long-term personality traits. For instance, as mentioned in the “Multiplayer games” section, researchers have supported the selection of game types, whether cooperative or competitive, based on participants’ inherent personalities [41]. Moreover, most researchers suggest that effectively integrating personality inferences can greatly enhance the comprehensiveness of perception models [103]. Various methodologies, including questionnaire-based assessments, physiological signal processing, and behavioral measures, have been employed to infer and predict user personalities [105]. Zhao et al. [106] successfully predicted personality traits by extracting features from EEG signals and subjective ratings. Another factor that facilitates the adaptation of interaction styles is memory. This requires agents to retain past interactions with users and adapt to their routines and personal preferences. These adaptations make the human–agent interaction more personalized, enhancing user comfort, trust, and adherence. A simple example is the agent’s ability to remember the names of different users and address them correctly in subsequent interactions. However, the factor of memory has remained virtually unexplored, and there are still many challenges that need to be addressed in the future.

**Fig. 5.**
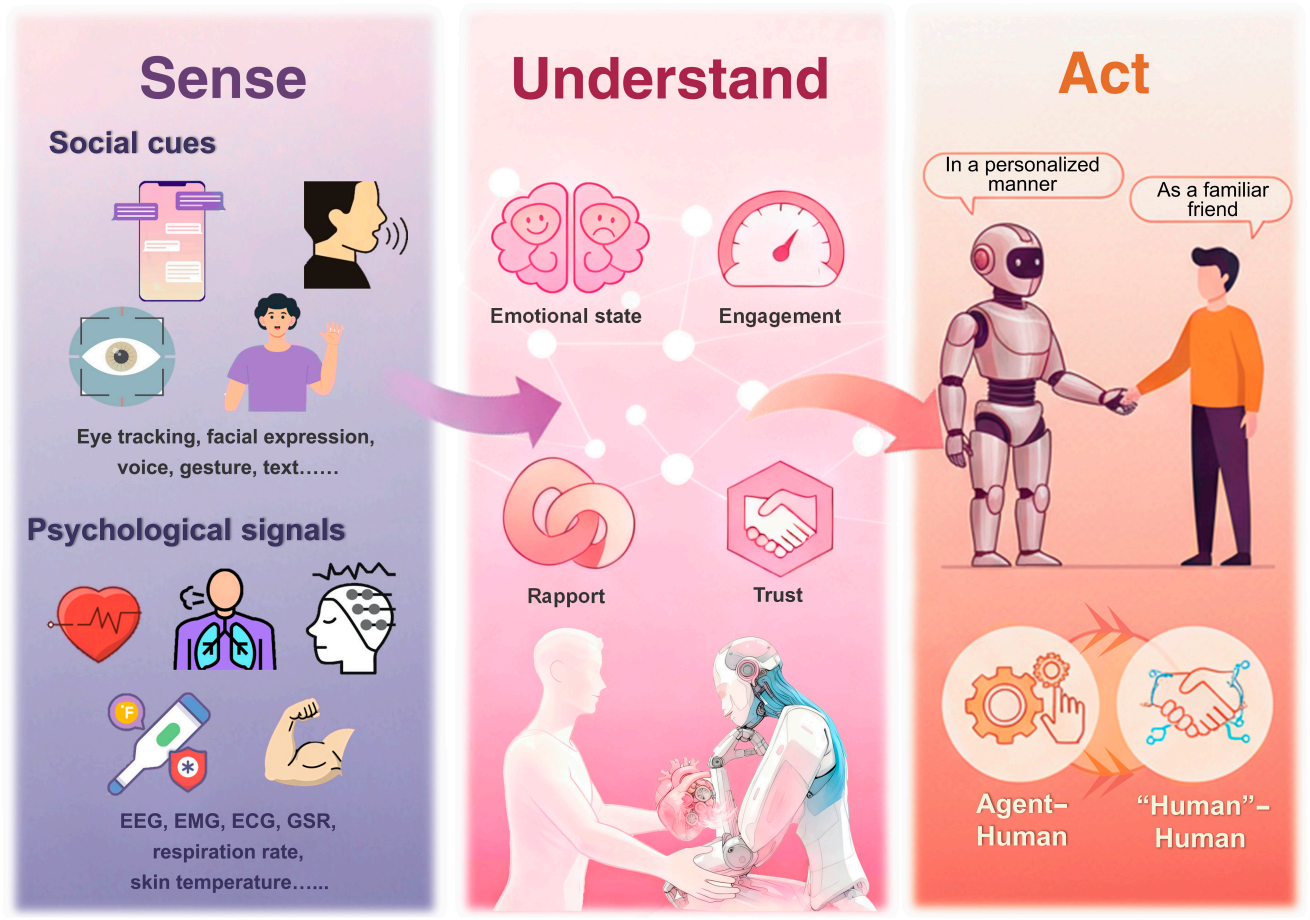
Multimodal sensing and interaction framework for future human–agent systems. This system integrates social and psychological signals to improve agents’ understanding and response to users’ emotions and behaviors, enabling deeper, more personalized, and familiar interactions. With enhanced artificial empathy, these agents could provide more effective interaction with users.

As perceptual and cognitive capabilities of agents continue to evolve, the ultimate goal is to realize genuine artificial empathy systems that can accurately perceive, understand, and respond to users’ physiological and psychological needs. In the healthcare domain, future agents could also be designed to selectively integrate relevant perceptual information that is tailored to their specific roles, including physicians, nurse practitioners, and psychotherapists, in order to enhance the depth and quality of interactions. Building on this trajectory, recent advances in GenAI have further expanded the possibilities. For example, Raza et al. [107] mapped pathways for GenAI in diagnostic and therapeutic tasks, while Luo et al. [108] introduced BiomedGPT, a vision-language model enabling diverse biomedical applications with greater efficiency and cross-modal understanding. Wang et al. [109] demonstrated that GenAI could be used to simulate emotionally supportive conversational agents in a healthcare-like scenario, showing promising user satisfaction and perceived empathy. Such systems illustrate the promise of more personalized and adaptive empathic interaction. However, practical deployments also reveal persistent barriers, including opacity in reasoning, high computational demands, and latency that compromise the fluidity of real-time exchanges [66]. Moreover, most generative models still lack emotion-aware evaluation in clinical contexts, limiting their ability to calibrate responses to patient needs. Fig. 5 describes an ideal interaction framework for future human–agent systems. Interactions between agents and humans are expected to transition toward deeper, more natural and seamless exchanges, thereby meeting the growing expectations for human-like engagement and enhancing artificial empathy.

## Critical Considerations of Artificial Empathy

While replicating the depth and nuance of human empathy continues to be challenging, notable progress has been achieved in recent years. However, its clinical and practical validation remains limited. As summarized in Tables 1 to 3, most studies employed small samples and short-term interventions, reducing the generalizability. The predominance of positive findings also raises concerns about publication bias and selective reporting. Collectively, these limitations underscore the need for more rigorous, large-scale, and longitudinal studies to establish robust evidence for the role of artificial empathy in healthcare [109]. Outcome measures across studies are also highly diverse. Most rely on self-report questionnaires, which provide useful insights but limit comparability across platforms. Physiological indices such as GSR, HR, and ECG have been proposed as more objective markers [32], but their applications in clinical trials remain scarce. This methodological heterogeneity complicates comparison across platforms and makes synthesis difficult. Another concern is the limited use of appropriate control conditions. Most existing studies compare intervention groups only with baseline performance, which makes it difficult to determine whether observed benefits are attributable to artificial empathy or to general therapeutic engagement. Therefore, future research should establish unified frameworks, integrate multimodal assessment indicators, and implement rigorous control designs to better guide the development and evaluation of artificial empathy technologies in clinical practice [110].

Beyond methodological concerns, recent analyses further highlight the ethical problems of embedding artificial empathy into healthcare. First, excessive reliance on the pseudo-empathy simulated by artificial systems may foster patterns of misuse and even false attachment [111]. Future developments in artificial empathy are expected to emphasize increasing personalization. Hence, interactions with robots and virtual agents risk becoming more appealing than real human relationships. Such displacement of authentic social interaction could undermine psychological well-being. Artificial empathy should therefore remain an adjunct, designed to support rather than to replace interpersonal communication. Its use is best confined to contexts of genuine necessity, such as mitigating workforce shortages in healthcare. Second, even as the most advanced systems simulate empathic interactions, GenAI continues to face the problem of hallucinations; false or misleading content can misguide users and cause serious harm, particularly in healthcare where accuracy is critical [112].

## Conclusion

In response to the global healthcare workforce shortages, advanced technologies are being developed to assist or replace certain human roles. However, integrating elements of human interaction, such as empathy and emotional support, remains a significant challenge. Interpersonal interaction plays a vital role across various domains, such as healthcare, education, services, and industry. The integration of these interactions can be achieved or simulated through a range of technical approaches. Multiplayer games offer a powerful method to build connections between users, even when they are geographically separated. These games are widely used among individuals undergoing physical rehabilitation or other forms of physical training, substantially enhancing their engagement and motivation. While direct interpersonal interaction offers the most effective social support, limited human resources often make it difficult to pair users with suitable partners. Therefore, exploring virtual interpersonal interactions presents a promising solution to address this challenge. Social robots and virtual agents offer ways to simulate interpersonal interaction as embodied and nonembodied forms, respectively. They aim to establish social rapport with users by employing various cues, including appearance, behavior, facial expressions, touch, and voice. They have demonstrated benefits for both mental and physical health. However, due to current limitations in technology, they often fall short of convincingly replicating human interactions. This may result in the uncanny valley effect or simply fail to meet user expectations.

The rapid advancement of AI technologies, especially LLM, presents a great opportunity to accelerate the anthropomorphization of artificial agents, allowing them to better comprehend human behavior and dynamically adapt to individual personalities and emotional states in real time. In addition, the continuous advancements in high-precision mechanical systems and sophisticated control models, coupled with the ongoing development of immersive digital platforms such as VR and AR, greatly elevate the execution capabilities of artificial agents. However, these advancements alone are insufficient. Greater emphasis should be placed on developing artificial empathy to enable deeper, more meaningful interactions between humans and machines. By improving agents’ ability to perceive and respond to both the physical and psychological needs of users, we can move closer to creating technology that truly complements human connection and care. Meanwhile, it is essential that the development of these technologies adheres to ethical and legal standards. Regardless of how sophisticated interpersonal interaction simulation technologies become, they should never be misused. Their purpose is to augment and support healthcare professionals, especially in resource-constrained areas, rather than replace the uniquely human elements of care, empathy, and judgment.

## Data Availability

No new data were created or analyzed during this study. Data sharing is not applicable to this article.
